# “What kind of man gets depressed after having a baby?” Fathers’ experiences of mental health during the perinatal period

**DOI:** 10.1186/s12884-021-03947-7

**Published:** 2021-06-29

**Authors:** Sarah Hambidge, Amy Cowell, Emily Arden-Close, Andrew Mayers

**Affiliations:** Department of Psychology, Bournemouth University, Poole, BH12 5BB UK

## Abstract

**Background:**

To date, information and support has been focused on mothers, with evidence that healthcare professionals overlook fathers’ mental health, and that fathers receive little or no support for themselves during the perinatal period. However, recently, fathers’ mental health has become an area of interest.

**Methods:**

This study explored the support fathers receive for their own mental health during the perinatal period. A qualitative questionnaire was distributed on social media networks and completed by twenty-nine fathers.

**Results:**

Thematic analysis produced three main themes: Factors Influencing Fathers’ Mental Health, Consequences of Poor Mental Health in Fathers and Solutions to Improve Fathers’ Mental Health.

**Conclusions:**

The findings from this study highlighted important implications about fathers’ mental health and the need to support them more effectively. Fathers’ reluctance to seek support and the limited support available need to be addressed. Fathers in this study perceived that perinatal health professionals view ‘mothers as the priority’. It is clear that health professionals need more training on how to recognise that fathers are also important and need support for their mental health.

**Supplementary Information:**

The online version contains supplementary material available at 10.1186/s12884-021-03947-7.

## Background

Becoming a father is an extremely important life event for a man [1]. Fathers can experience new emotions, feelings and changes initiated by the transition into parenthood [2]. While there is a relative abundance of research focusing on maternal mental health, data on fathers is still limited by comparison. A systematic review of 18 studies confirmed increased paternal stress in the perinatal period, especially around the time of birth [3]. The increase in stress had an impact on other mental health problems for those fathers. Estimates of paternal mental health problems differ depending on the characteristics of the sample and the method of measurement [4]. Some evidence shows that fathers can struggle with mental health problems before, during and after pregnancy [5]. A meta-analysis of 43 studies suggested 10.4% of new fathers experience depression compared to 4.8% of men from the general population [6]. Paternal postpartum depression is prevalent in 4–25% of new fathers during the first 12 months after birth, but due to a lack of awareness and recognition of symptoms prevalence is likely underreported [7]. Estimated rates of depression range from 24 to 50% for men with partners who experience maternal postpartum depression in the 12 months after birth [8]. However, during their partners’ pregnancy, only 3.2% of 2000 fathers reported seeking help for depressive symptoms [9], in comparison to 13.6% of women [10].

In recent decades, men have been taking a more active role in childbirth [11]. The presence of fathers at the birth has increased within the last few decades, with around 98% now reported as attending childbirth [4]. A father’s involvement can have a positive impact on maternal well-being and coping abilities [12, 13], pregnancy outcomes [14], parental roles [15] and the child’s continued physical, mental, behavioural, social and emotional development [2, 16]. However, healthcare professionals have traditionally overlooked men’s mental health during the transition to parenthood [17]. Other research confirms that fathers perceive a lack of desired support for their mental health from healthcare professionals [18]. Healthcare systems such as the United Kingdom (UK) National Health Service (NHS) have primarily focused service provision on maternal mental health and wellbeing during a woman’s transition into parenthood. Our recent research has confirmed that fathers feel their mental health is overlooked by perinatal health professionals [19, 20].

Research suggests receiving appropriate support from healthcare professionals during these critical periods reduces fathers’ risk of psychological distress [21]. However, during childbirth many fathers have reported being ignored by healthcare professionals and feeling invisible, uninvited and uncomfortable [13, 22, 23]. Focusing on data from 2013, Massoudi reported that less than one in five nurses offered any type of support to fathers during the perinatal period [24]. Fathers who had lost a baby felt that they were simply seen as ‘there to support the mother’ rather than being treated as the father with their own needs [25]. A recent review examined 46 studies focusing on fathers who had experienced the loss of their child in pregnancy or at birth [26]. Fathers in these studies consistently reported poor understanding and support following such loss. These findings reinforce the notion that the impact of parenthood on fathers is largely overlooked [27].

Gender-specific factors relating to support seeking have been identified. Better mental health literacy is strongly associated with more help-seeking behaviour [28]. Men typically have poorer mental health literacy than women [29]. Fathers are more likely to hide mental health issues during pregnancy and the perinatal period [30–32] because of societal pressure to support their family emotionally and financially [27]. Men typically do not expect any support for themselves during antenatal classes, perceiving their role as to support the mother [20]. Further, during the perinatal period fathers report being unaware of how or where to seek mental health support [17]. If fathers do not anticipate receiving support, they are less likely to know support exists and unlikely to initiate their own support seeking regarding their mental health.

The reported lack of support and barriers to support seeking, combined with a sense of feeling excluded by healthcare professionals have caused fathers to experience conflict between the expectations and realities of fatherhood during the perinatal period [33, 34]. Fathers expect to have a primary role during pregnancy and birth but lack awareness and confidence regarding how to involve themselves [35]. This is influenced by the lack of information and support fathers are provided with to help guide them through the transition into parenthood, leaving them with challenging realities [36]. Where interventions are developed to fathers’ mental health, it is not always clear how well fathers will engage in those services. Recent research from Australia identified several factors that help identify barriers that may prevent fathers from participating in support programmes [37]. The authors concluded that more attention is needed to encourage motivation and make these services more accessible.

It is also important to consider the impact of poor mental health on fathers in the perinatal period. Suicide rates are higher in men than women, possibly due to reduced support seeking [38], the most common cause of death in men aged under 50 is suicide [39], and men who experience symptoms similar to those of postnatal depression are 47 times more likely to be a suicide risk [40]. Fathers’ poor postnatal mental health also significantly impacts the family, especially the infant, being related to greater risk of child behaviour problems at 3½ years [41] and conduct problems at 7 years [16]. Research that focuses on the support fathers feel that they receive for their own mental health is therefore needed. Some regional UK charities that already support mothers also now have support groups for fathers (such as Bluebell Care in Bristol, UK), but this is rare.

This research aimed to explore fathers’ perceptions of the support they received for mental health problems during the perinatal period. A more in-depth understanding of fathers’ experiences means barriers to support seeking for mental health problems can be identified, leading potentially to suggestions of possible ways to break down those barriers and offer improved support.

## Method

### Sample

Fathers were included in the study if they at least 18 years old, where their child was born in the last 10 years, and they had a history of poor mental health during, or after, their partner’s pregnancy. These inclusion criteria were used for screening for participation. Participants were asked to specify the nature of those symptoms as part of the study questions. Mental health challenges disclosed included depression, anxiety, stress, borderline personality disorder, bipolar disorder and obsessive-compulsive disorder. Forty-five fathers initially participated in the study; data from 29 of these was analysed.

#### Recruitment

Potential participants were invited via social media advertising (Twitter, Facebook and LinkedIn), which targeted fathers who had experienced mental health problems during the perinatal period. The social media expressly tagged mental health support groups for fathers, such as Make Birth Better and Dad Matters UK. The advert included a link to access information about the study, via Qualtrics™. Potential participants were required to answer screening questions. Only if those questions were answered positively could they indicate consent. All participants were adult fathers who stated that they had experienced poor postnatal mental health. Once they had indicated consent, the participants could access the survey, which they completed online. Demographic information is shown in Table 1. All of the fathers were living in the UK, were married, cohabiting or single at the time of study, and had been present at the birth of their child.

**Table 1:** Demographic information

Demographic information	Categories	Number of respondents
Age of Participant	18–24 years old	4
	25–34 years old	12
	35–39 years old	4
	40–49 years old	4
	50 years old or over	2
Relationship Status	Married	18
	Co-habiting	12
	Single	4
Ethnic Group	White British	16
	White Irish	4
	Black British	2
Gender of Child	Female	8
	Male	12
Father involvement during perinatal phase	Yes	29
	No	0

#### Procedure

A qualitative questionnaire (see Additional file 1) containing twelve questions was created based on guidance from experts in fathers’ mental health, including the corresponding author and leading UK campaigners such as Mark Williams. Using qualitative questionnaires is a well-established method for gathering information about sensitive topics, as it maintains participant anonymity [42]. Topics covered in the questionnaire included the participants’ history of mental health, mental health status during pregnancy, birth and postpartum, diagnosis and support received and relationship changes with partner and child as their mental health status changed. The questions sought to gain a better understanding of fathers’ mental health during the perinatal period with a specific focus on their awareness of their own mental health, and behavioural changes including support seeking and if they received support. The questionnaire was distributed through Qualtrics®, an online portal. The study focused on potentially sensitive topics around mental health, which many of the participants may not have spoken about before. To reduce potential experiences of distress, participants were told to write only what they were comfortable with sharing. Participants were also signposted to sources of support before and after completion of the questionnaire. Responses were kept anonymous to protect confidentiality and give participants the chance to speak freely about their experiences.

#### Data analysis

Data was analysed using thematic analysis [43] to allow detailed exploration of the responses. Analysis began with familiarisation of the data leading to the development of initial ideas and potential prominent codes. Initial codes were then collated to identify potential themes and sub-themes. Themes identified were reviewed and some of the initially identified patterns merged to form broader themes. The initial data set was re-read to ensure the themes were representative of the data. Thirteen cases (approximately half the sample) selected at random were second coded and the overall authenticity of the themes checked and verified to ensure good inter-rater reliability. Although Braun and Clarke [42] criticise inter-rater reliability on the grounds that it contradicts the interpretative nature of qualitative research, we felt that it was important to assess in this case as the responses provided limited opportunity for interpretation. Coding was an inductive and data-driven process, not informed by an a priori framework.

#### Ethics

The study was approved by Bournemouth University, Faculty of Science and Technology Research Ethics Committee (Bournemouth University Ethics identification number, 28659). Participants were informed that their participation was voluntary, and they had the right to withdraw without adverse consequences. Participants were provided with an electronic information sheet and invited to confirm consent to take part and provided with debriefing information. Anonymity was ensured through the removal of identifiers. All data were stored confidentially in accordance with the UK General Data Protection Regulations.

## Results

Eleven participants (38%) received a medical diagnosis for their mental health condition, while the remaining eighteen (62%) self-diagnosed. Participants’ reported (and in some cases, diagnosed) mental health problems included depression, anxiety, post-traumatic stress disorder, obsessive compulsive disorder, bipolar and borderline personality disorder. Five participants (17%) reported experiencing mental health problems prior to their partners pregnancy, eight participants (28%) during the pregnancy and sixteen participants (55%) for the first time during the postnatal period. There were also differences regarding duration of symptoms experienced and personal recovery from these symptoms. Some participants’ symptoms declined immediately after the birth of their child, whilst for others symptoms continued for up to a year or more.

Participants demonstrated varying levels of insight into their own mental health. They reported that their mental health was negatively influenced by changes in their relationship with partners; adjustments in lifestyle choices and the home environment and routine; and coping with pregnancy symptoms and complications and the arrival of a new baby. Those who had experienced what they considered poor mental health prior to their partner’s pregnancy reported greater awareness of mental health symptoms, whereas participants with no prior experience of suspected mental health problems required information regarding the signs of mental health problems.

**Fig. 1:**
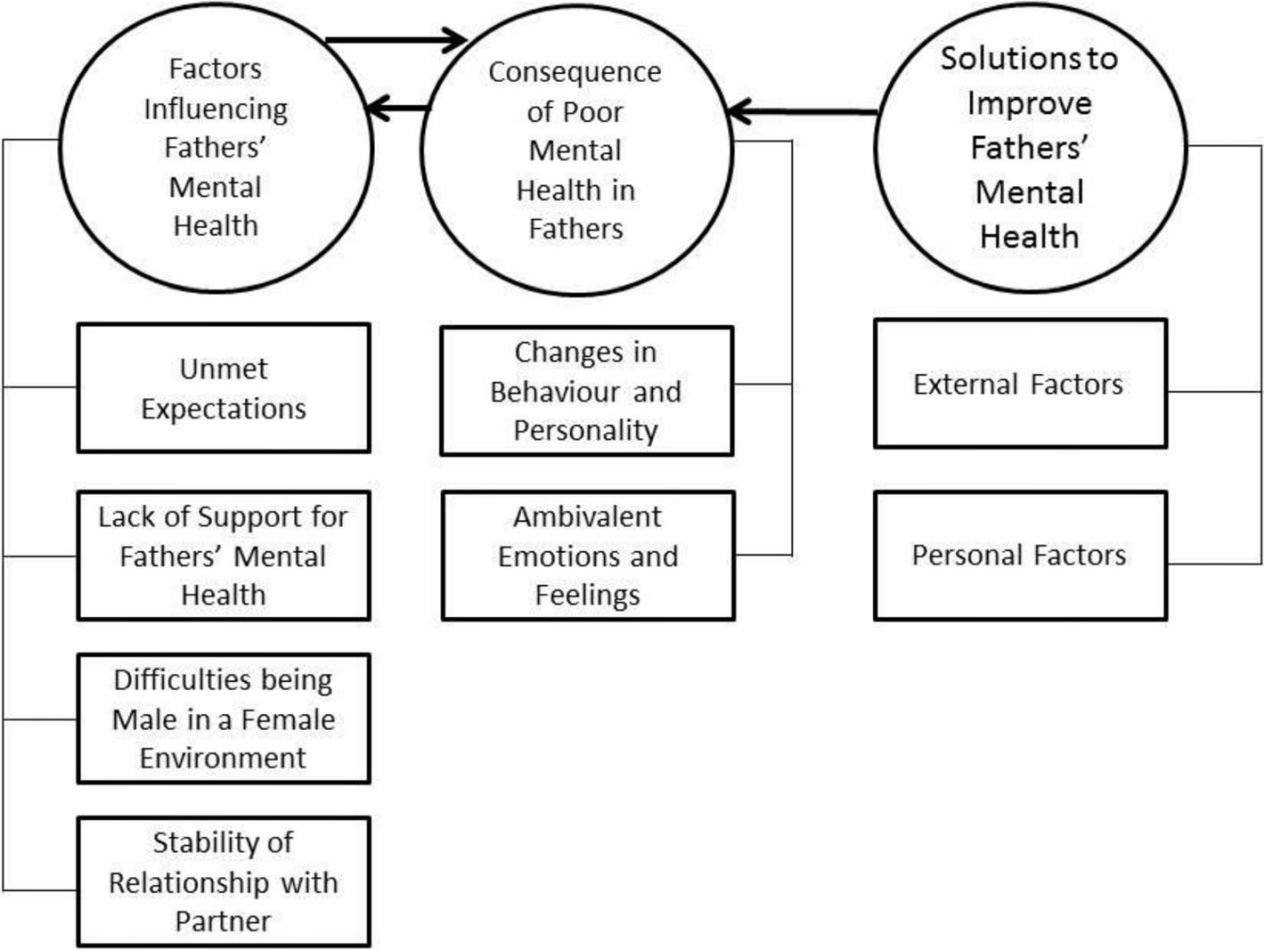
Themes and sub-themes identified

Thematic analysis identified three key themes: “Factors Influencing Fathers’ Mental Health” (Sub-themes: “Unmet Expectations”, “Lack of Support”, “Significance of Mothers During the Perinatal Period” “Stability of Relationship with Partner”); “Consequences of Poor Mental Health in Fathers’” (Sub-themes: “Changes in Behaviour and Personality”, “Ambivalent Emotions and Feelings”); and, “Solutions to Improve Fathers’ Mental Health (Sub-themes: “External Factors”, “Personal Factors”). These themes are illustrated in Fig. 1.

### Factors influencing fathers’ mental health

#### Unmet expectations

The realities of pregnancy and the postpartum period differed significantly from participants’ expectations. One participant described fatherhood as: *“not just changing nappies and night feeds”* (P2). The gap between expectation and reality caused him to believe he was failing as a father and a husband: *“I went from being able to try and be super-dad for my first son, to not feeling like I could do anything for either of the boys”* (P2). Another expected fatherhood to be a positive time but found that coping with a new baby left him wondering *“whether we would ever have a happy time”* (P5). Participants said they would advise future fathers on the realistic expectations of becoming a father and encourage men to research what parenthood entails as: *“it’s harder than you could possibly imagine”* (P8).

#### Lack of support for fathers’ mental health

Fifteen participants (some of whom did not receive a diagnosis of a mental illness) claimed they were not offered any support from health care professionals “*explicitly for mental health”* (P4) during the perinatal period. This referred to potential as well as actual mental health problems. They expressed the view that healthcare professionals were unconcerned about fathers’ mental health, and support is only offered once *“you try to harm yourself or you have a breakdown”* (P1). For example, one participant who witnessed his partner’s complicated birth of their premature son: *“wasn’t offered any help or support”* (P9) to cope with the traumatic event. Lack of support aimed at preventing mental health problems left participants feeling uncertain and lacking confidence in their role as a father: *“(I) didn’t know how things would turn out”* (P6).

#### Difficulties being male in a female environment

Many participants experienced difficulties being male in a female focused environment which affected their sense of masculinity, leading them to question their role as a man. One participant claimed he *“felt like a failure, no true man”* (P11). One father asked *“what sort of man gets depressed after they’ve had a baby?”* and described feeling *“lots of shame”* as a result of his mental health diagnosis (P3). Nine participants described the lack of support they received in comparison to support their partners received: *“my wife had plenty [of support]”* (P8), *“[their] focus is on mothers”* (P6). One participant reported feeling overshadowed by the support his partner received:

*“Everyone seemed to be there for her … the midwives in the hospital barely speak to the father. One [midwife] offered me a coffee, but that was literally it. Fathers are pretty much ignored throughout the process”* (P8)*.*

One participant reported receiving information on how to support his partner when she developed postnatal mental health problems, but no information about how to seek support for himself: *“looking back the institutions, family and myself, focussed on how I would support my wife and the emphasis was on me staying strong”* (P5). During their engagement with these services (i.e. antenatal classes, birth of the baby), participants were unclear regarding their role, and felt there was an expectation from healthcare professionals that they should support their partner.

#### Stability of relationship with partner

Nineteen participants said their perceived or actual mental health problems negatively affected their relationship with their partner during the perinatal period. The relationship difficulties developed from the rapid changes parents experienced preparing for and/or welcoming a new baby into their lives including difficulties associated with adapting to a new routine: *“The relationship was much more strained after the birth due to the pressures relating to lack of sleep and exhaustion”* (P16). Participants reported breakdown in communication, increased arguments and spending less time with their partners. All participants agreed the importance of trying to maintain a stable relationship with their partner during the perinatal period for the benefit of their partner and the baby’s wellbeing: *“Rest and spend as much quality time as you can with your partner before the birth. Do all you can to reduce work commitments”* (P4).

### Consequences of poor mental health in fathers

#### Stability of relationship with partner

Poor paternal mental health was a significant factor contributing to relationship difficulties. For example, one participant reported being signed off work due to his mental health diagnosis, which in turn caused problems with establishing a new routine with the baby and impacted his mental health further: *“my wife couldn’t establish a routine [with the new baby] which compounded my depression as I felt like I wasn’t just failing with fatherhood, but husbandhood”* (P5). Only four participants, who developed support systems with their partners, described their perceived mental health problems as having a positive impact on their relationship: *“very supportive … she encouraged me to open up”* (P11).

#### Changes in behaviour and personality

Changes in their behaviour and personality created challenges for participants throughout the perinatal period. They reported a lack of control over situations, experiencing feelings of failure, lack of confidence and low self-esteem: *“I struggled with anxiety and intrusive thoughts. After the birth this got significantly worse to point I couldn’t leave my bedroom. It severely impacted the relationship with my partner and missed out on opportunity to bond with baby.”* (P27). A significant challenge for participants was their perceived ability to be a parent, with many focusing on their limitations rather than their successes: *“it [perceived mental health problems] reduced my ability to deal with situations that would have otherwise been manageable.”* (P1). The perceived inability to be a “good” parent caused a fragmentation in the relationship and their ability to bond with their child: *“It affected my bond with my daughter, which sadly I still feel now. I don’t feel the same feelings towards her as I do to my first child, which makes me sad”* (P3). Participants felt their behaviour and personality changes contributed to relationship problems due to expressing new and heightened emotions which were absent prior to pregnancy:

*“[Changes in behaviour] … lead to a number of arguments, a feeling of resentment on my part as there seemed to be nothing but worry and anxst [angst] at what I was hoping was going to be a joyous time” (P5).*

This consequently impacted how they perceived their mental health problems further: *“I cried for the first time ever. Just burst into tears feeling I wasn’t good enough”* (P10).

#### Ambivalent emotions and feelings

An overwhelming feeling of low mood and uncertainty was common during the perinatal period. All participants recalled feelings of doubt and stress which triggered either diagnosed episodes of depression (*n* = 14) or perceptions of low mood (*n* = 15):

*After the birth it would have been nice if I would have been referred for specialist mental health care prior to thoughts of self-harm … My partner supported me as much as possible but obviously her priority was our son. I felt scared that she would leave. Obviously, the stress and anxiety exacerbated things. I went from being pretty active to spending 22 h a day in bed (P27).* These episodes resulted in three participants experiencing suicidal thoughts: *“It was a crisis point where OCD was making me suicidal”* (P6).

### Solutions to improve fathers’ mental health

#### External factors

Participants suggested a variety of potentially helpful support mechanisms for understanding and dealing with potential mental health problems. These ranged from health care professionals *“asking how dad is”* (P7) and *“acknowledging that he [dad] was there” (P4),* through to *“practical, behavioural, situational post-natal classes and workshops”* (P2) and *“practical help with keeping the home running”* (P10). Several participants suggested longer paternity leave could provide men additional time to adjust to the arrival of their baby, and potentially mitigate some factors contributing to possible mental health problems.

#### Personal factors

One participant advised future fathers to: *“avoid macho dads, find other men who are able to express some feeling and that can relate to the load, [the] stress and [the] difficulties that lie ahead”* (P1). Participants suggested future fathers should be realistic about coping with the transition into parenthood and the support they can provide their partner and child: *“don’t set your parenting bar too high [because] this may negatively affect mental health”* (P2). They felt new fathers should be made aware of the potential to experience these types of feelings and that it is acceptable for men to express their emotions and to seek support if they feel they are struggling to cope: *“don’t be afraid to ask for help and accept your limitations”* (P10).

## Discussion

Fathers had varying degrees of awareness of the symptoms of mental illness. Fathers’ believed their risk of experiencing mental health problems during the perinatal period was influenced by a range of external factors including unmet expectations, lack of support for fathers’ mental health, difficulties being male in a female environment and the stability of their relationship with their partner, and had significant consequences for fathers including uncertainty and changes in both personality and behaviour. Both external and personal solutions to help fathers with their mental health were suggested. Pregnancy was the most common time period for fathers to develop mental health problems and fathers’ responses suggested that the anxiety of preparing for the baby during pregnancy contributed to increased levels of stress. Previous studies also found pregnancy to have a negative effect on fathers’ stress [3] and mental health [27].

Fathers’ perceptions about their own mental health was influenced by how they believed they were perceived by healthcare professionals. Most participants were unaware fathers could suffer perinatal mental health problems, often questioning the legitimacy of their own experiences. This may be because perinatal healthcare professionals are often unaware of fathers’ mental health; Massoudi [24] reported that most nurses failed to recognise or attend to fathers’ emotions and distress during the birth. However, in general men are less likely than women to recognise their mental health problems [44, 45], and subsequently less likely to discuss them. Although men are twice as likely as women to have low health literacy, which can affect acknowledgement of health problem and influence subsequent support seeking behaviour, it is not clear why this gender difference occurs [31]. It may be men are less likely to discuss mental health problems due to gender specific role socialisation. The man’s role includes maintaining emotional control and physical strength. Fathers indicated their unwillingness to accept or discuss their mental health problems may have been due to compliance to gender norms and maintaining the focus of support on their partner and baby [46].

This study indicated how fathers perceived that healthcare professionals lacked awareness regarding their mental health. This may lead fathers to question the validity of their mental health problems [17]. Many of the fathers in this study reflected on how they were overlooked by health professionals regarding their mental health. The theme around ‘being a male in a female environment’ illustrated how fathers perceived a need for some focus on their wellbeing. Some fathers suggested that health professionals would only consider supporting their mental health if they were at risk of harm. This lack of recognition from health professionals further reduced fathers’ confidence to seek help. Our findings regarding health professional support for fathers’ mental health has also been confirmed in recent research (e.g. [18, 37]).

Fathers reluctance to seek support for their mental health problems was reinforced by their perception that when engaging with maternity services they were in a mother-orientated environment. Indeed, this may question whether the term ‘maternity services’ should be replaced with ‘pregnancy services’ to widen the focus to both partners. Their mental health problems led to them questioning their masculinity, indicating norms of stigma around mental health issues in men. Previous research also suggests that fathers are influenced by “perceived expectations of masculinity” [17]. Men have expressed the need for father-only antenatal classes [47], indicating their willingness to learn more about supporting their partner and coping in the perinatal period. Such classes might subsequently provide fathers with added confidence regarding their roles when in mother-dominated environments (i.e. during birth), leading to being a man in a female experience becoming a less prominent risk factor for fathers’ mental health problems. However, any intervention and support for fathers’ mental health needs to consider existing evidence around fathers’ perceived barriers to accessing these [37]. Further research into conformity to masculine norms is necessary to reduce stigma towards men and subsequently help fathers become more accepting of their mental health problems in female-dominated environments [48]. Further, fathers mentioned difficulties around going back to work after the birth and requesting extended time off work. Paternity leave in the UK is significantly shorter than other countries (such as Sweden [49]), which may exacerbate fathers’ mental health problems.

In common with previous research suggesting that fathers experienced negative emotions such as loss of control due to the unexpected realities after the birth [50], differences between expectations and realities were identified. In the present study, fathers exhibited feelings of isolation and anxiety regarding their parenting abilities. Most fathers reported not researching mental health prior to the birth of their baby, as they had no reason to expect that they would develop problems. Fathers who conducted research (typically those with previous history of mental health problems) noted that available information focussed on women, which for them questioned the legitimacy of their experiences. Previous research identified that expectations of birth are dependent on the information and support fathers receive from healthcare professionals [51]. Similarly, in the present study, fathers received minimal or no support during the perinatal period which may have contributed to the discrepancy between expectation versus realities and subsequent mental health problems [36]. Our findings regarding the lack of support provided for fathers aligns with previous literature indicating fatherhood is overlooked in research and by healthcare professionals [25, 33, 52]. For example, despite making ample consideration for maternal mental health, the National Institute of Clinical Excellence (NICE) guidelines do not make any provision for fathers [53].

Fathers described the limited support they received in comparison to their partners. Many recognised they are viewed as a support mechanism and that the mothers’ wellbeing is the primary focus for healthcare professionals [25, 54]. Whilst fathers appreciated that the mother is the priority, this caused them to question their role in the perinatal period, leading to feelings of isolation. Similarly, Widarsson et al. [23] reported that fathers felt excluded during pregnancy. Fathers desire to be perceived as an individual rather than simply a support mechanism for the mother [55].

Most fathers in this study reported that their mental health problems put a strain on their relationship. Becoming parents acts as an amplifier for current problems in a relationship, thus resulting in difficulties maintaining a stable relationship [56]. The fathers in this study also reported experiencing behaviour and personality changes as a consequence of their mental health, and difficulty bonding with their children in some cases, often questioning their parenting ability. This is important as previous research indicated fathers’ mental health has a significant impact on a child’s development [16]. Participants experienced heightened emotions as a result of becoming a father and suffering with mental health problems. Fathers have reported more positive emotions in previous research [2]. However, in the present study, fathers expressed negative emotions and feelings such as anxiety, stress and feeling like a failure, possibly due to the anonymity of the questionnaire. Fathers need to be recognised and educated by healthcare professionals, from health visitors to midwives, with a specific focus on mental health. Recognition should be aligned with support for fathers throughout the perinatal period.

### Strengths and limitations

This study added to the limited knowledge that we have about how fathers perceive their mental health in the perinatal period and addressed some aspects regarding the support that they get for that However, there are some limitations that need to be considered. While a qualitative questionnaire was seen as the most appropriate method of data collection due to the sensitivity of the topic, the responses participants provided were not very detailed and it was not possible to explore responses in more depth. Related to this, the wording of the questions could have influenced participants to respond more negatively than might actually be the case, as the questionnaire focused on mental health difficulties rather than positive aspects of mental health. Furthermore, although the brevity of the fathers’ responses could be seen as a limitation, it might also be further evidence of their reticence to talk openly about their mental health. A sample of 29 participants is relatively small for a qualitative questionnaire. Mental health problems were self-reported. However, the fact that 18 participants self-diagnosed as having mental health problems furthers our argument that fathers are reluctant to seek help for their mental health problems. Finally, we did not require participants to report their sexuality. Future research may need to account for this, as outcomes may vary between heterosexual and non-heterosexual men. We were mindful of attracting a diversity of participants, which is reflected in the image that we used in the study advertisement.

## Conclusion

A large proportion of fathers experienced mental health issues during the perinatal period. Fathers perceived that they receive inadequate support for their mental health issues relative to mothers. Although fathers acknowledged that the mother should be put first, they need to acknowledge and support their own mental health. However, this is unlikely if they continue to be overlooked during the perinatal period by health care professionals and services.

Fathers needed to be taken into account during the perinatal period, and made aware that support exists for men, to reduce the opportunity of developing mental health problems in both parents. This may start by identifying ways to increase help seeking in men, which may be additionally enhanced by identifying ways to improve men’s mental health literacy. There is also a need to enhance awareness regarding the importance of fathers for family wellbeing and providing adequate support for both mother and father. This could be facilitated by increasing the capacity and awareness within health professionals and the service system.

## Supplementary Information

**Additional file 1.**

## Data Availability

The datasets used and/or analysed during the current study are available from the corresponding author on reasonable request.

## Abbreviations

UK: United Kingdom
NHS: National Health Service
