# Supplementary material for: “What kind of man gets depressed after having a baby?” Fathers’ experiences of mental health during the perinatal period
Source: BMC Pregnancy Childbirth. 2021 Jun 29;21:463. doi: 10.1186/s12884-021-03947-7 (PMC8244226; doi:10.1186/s12884-021-03947-7)
Supplement: Supplementary file 1 — Additional file 1. [file 12884_2021_3947_MOESM1_ESM.docx]

**Appendices**

Appendix 1: Questionnaire

1. How was your mental health during the pregnancy, labour and after the birth?

2. Do you have a history of mental illness?

3. Were you diagnosed at any stage (of the pregnancy and postpartum) with a mental illness?

4. What support did you feel you received during pregnancy?

5. What support did you receive after the baby was born?

6. What support would you liked to have had?

7. How much did you search your own information regarding antenatal depression or postnatal depression (especially in fathers)?

8. When did you first start to consider your own mental health and why?

9. How did the relationship between you and your partner change after the birth compared to how it was before?

10. To what extent did this affect your mental health?

11. Did your behaviour change during pregnancy and/or after birth? If so, how?

12. What advice would you give to first time fathers transitioning into parenthood?
